# ResNet-based image processing approach for precise detection of cracks in photovoltaic panels

**DOI:** 10.1038/s41598-025-09101-z

**Published:** 2025-07-08

**Authors:** Montaser Abdelsattar, Ahmed AbdelMoety, Ahmed Emad-Eldeen

**Affiliations:** 1 Electrical Engineering Department, Faculty of Engineering, South Valley University, Qena, 83523 Egypt; 2 Renewable Energy Science and Engineering Department, Faculty of Postgraduate Studies for Advanced Sciences (PSAS), Beni-Suef University, Beni-Suef, 62511 Egypt

**Keywords:** Crack detection, Electroluminescence, Image processing, Photovoltaics, Energy science and technology, Engineering

## Abstract

Advancing renewable energy solutions requires efficient and durable solar Photovoltaic (PV) modules. A novel mechanism based on Deep Learning (DL) and Residual Network (ResNet) for accurate cracking detection using Electroluminescence (EL) images of PV panels is proposed in this paper. Different kinds of ResNet architectures, where ResNet34, ResNet50, and ResNet152 were tested, came out with an F1-Score of 86.63%, 87.37%, and 88.89%, respectively. Although the accuracy for ResNet152 is slightly higher, ResNet34 was chosen as the best model since it gives us a trade-off between detection performance and computational performance. The main contribution in this research is the design of an efficient crack detection system trained on a large PV power dataset composed of 2000 EL images collected from different polycrystalline and monocrystalline cells. Although the dataset has some imperfections, to guarantee the presence of many cell states in each subset, it was split into training (70%), validating (20%), and testing (10%). This research demonstrates the application of advanced DL frameworks for early defect diagnosis from raw data to enhance PV panel maintenance, thereby bolstering the sustainability of solar systems. This research also has a significant impact on the academic industry, offering practical solutions for the renewable energy sector during periods of sustainable energy instability, particularly when new materials supplement PV panel usage. The technology preserves the efficiency of solar modules and encourages clean energy solutions by accurately identifying PV panel faults. The study lays a foundation for the further development of image-based defect detection methods in PV systems.

**Supplementary Information:**

The online version contains supplementary material available at 10.1038/s41598-025-09101-z.

## Introduction

The history of Photovoltaic (PV) technology goes back to 1839, when French physicist Edmond Becquerel discovered the PV effect. Since that time, PV technology has been used in applications from outer space exploration to powering the average residence^1^. PV technology is necessary for sustainable energy progress because it converts sunlight into clean and renewable energy as electricity, which decreases dependence on fossil fuels and reduces environmental impacts^2–4^. Although PV power is increasingly appealing, its intermittent nature and environmental dependency impose serious operational challenges. For example, dirt buildups on solar panels can lower power generation by a large percentage. With the growing integration of PV systems into classics, from iconic structures down to historical buildings, it will help with sustainability and going green^5^.

The upkeep of PV panels is crucial for multiple reasons. Firstly, it encompasses crucial tasks, such as cleaning and ensuring safety measures, which are necessary for preventing any decrease in the performance of the panels. Performing routine maintenance guarantees the seamless and effective functioning of the PV systems^6^. Consistent surveillance and upkeep are important to guarantee dependable energy generation from PV panels. This reduces the influence of environmental factors on energy production and preserves the durability of the solar panels.

The presence of cracks in PV panels can have a substantial effect on their overall performance and efficiency. Cracks in the panel cause a decline in the electricity output of the solar PV system, resulting in diminished overall efficiency. Cracks in Building-Integrated Photovoltaic (BIPV) modules can lead to a significant decrease of up to 43% in power output^7^.

The detection of cracks in PV panels is a difficult task, as PV panels are brittle and need careful inspection. Although these cracks are often detected using methods such as Electroluminescence (EL) imaging, advanced image processing techniques are needed for proper classification and quantification of the defects identified. Moreover, the diversity of possible types of cracks and their different influences on panel behavior renders exact identification difficult.

**Fig. 1:**
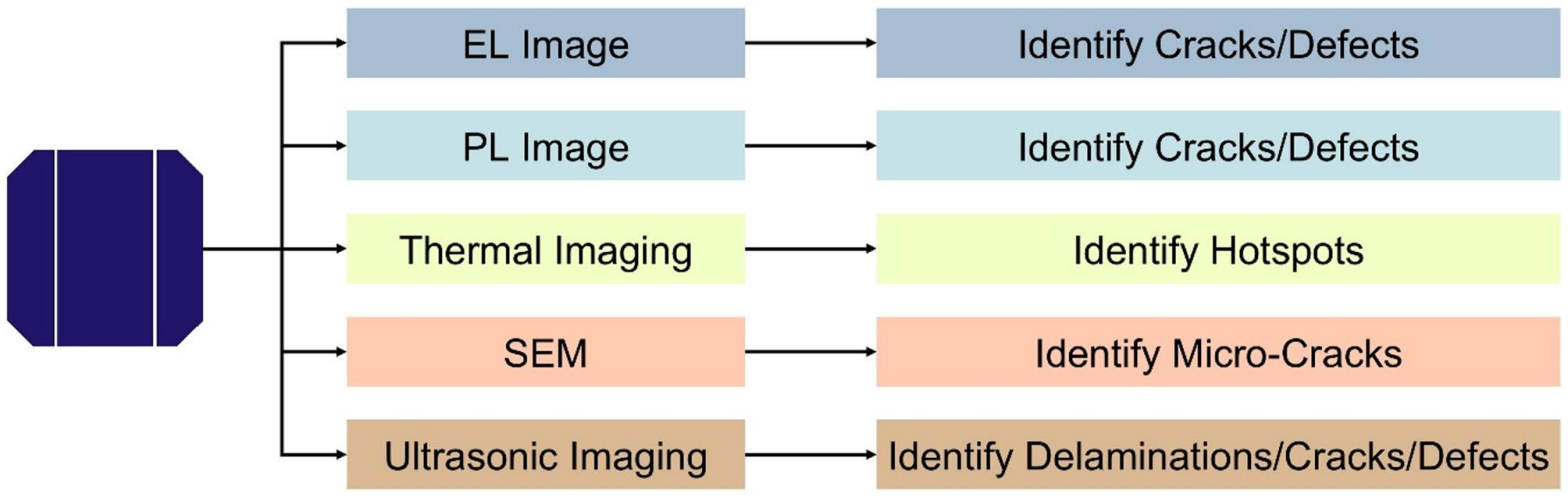
Overview of imaging approaches for identifying defects in solar cells.

Figure 1 illustrates a comparative overview of different solar cell defect imaging techniques. Each technique is useful for finding a specific type of defect. One of the most commonly used techniques is EL, which works while a current pass through a panel to make it emit light^8^. The emission of that light serves to highlight damage and defects, with cracks or other defects showing up as dark spots or lines on the image. In the same way, Photoluminescence (PL) imaging also uses light emission to sense cracks and defects, but with a different panel excitation and observation approach, it offers another powerful defect detection technique^9^. Another vital technique is thermal imaging, mainly employed to localize hotspots on the panel^10^. These hotspots are often due to cracks or partial delamination that affect the performance of the panel, and localized heating is a sign that it is not functioning well. Unlike the imaging methods based on the light emission. Using a focused electron beam, Scanning Electron Microscopy (SEM) provides high-resolution images of the panel surface that enable very fine fractures to be detected which may go unnoticed but over time could affect the efficiency of the panel. Ultrasonic imaging is used to identify various interior flaws, such as delamination, fractures, and other structural anomalies^11^. This approach detects subsurface faults by transmitting ultrasonic waves through the panel and measuring the returned signals. The integration of these modern imaging methods guarantees the accurate detection of flaws in solar panels, ranging from micro-cracks to significant structural issues, hence facilitating maintenance and enhancing efficiency in the renewable energy industry.

**Fig. 2:**
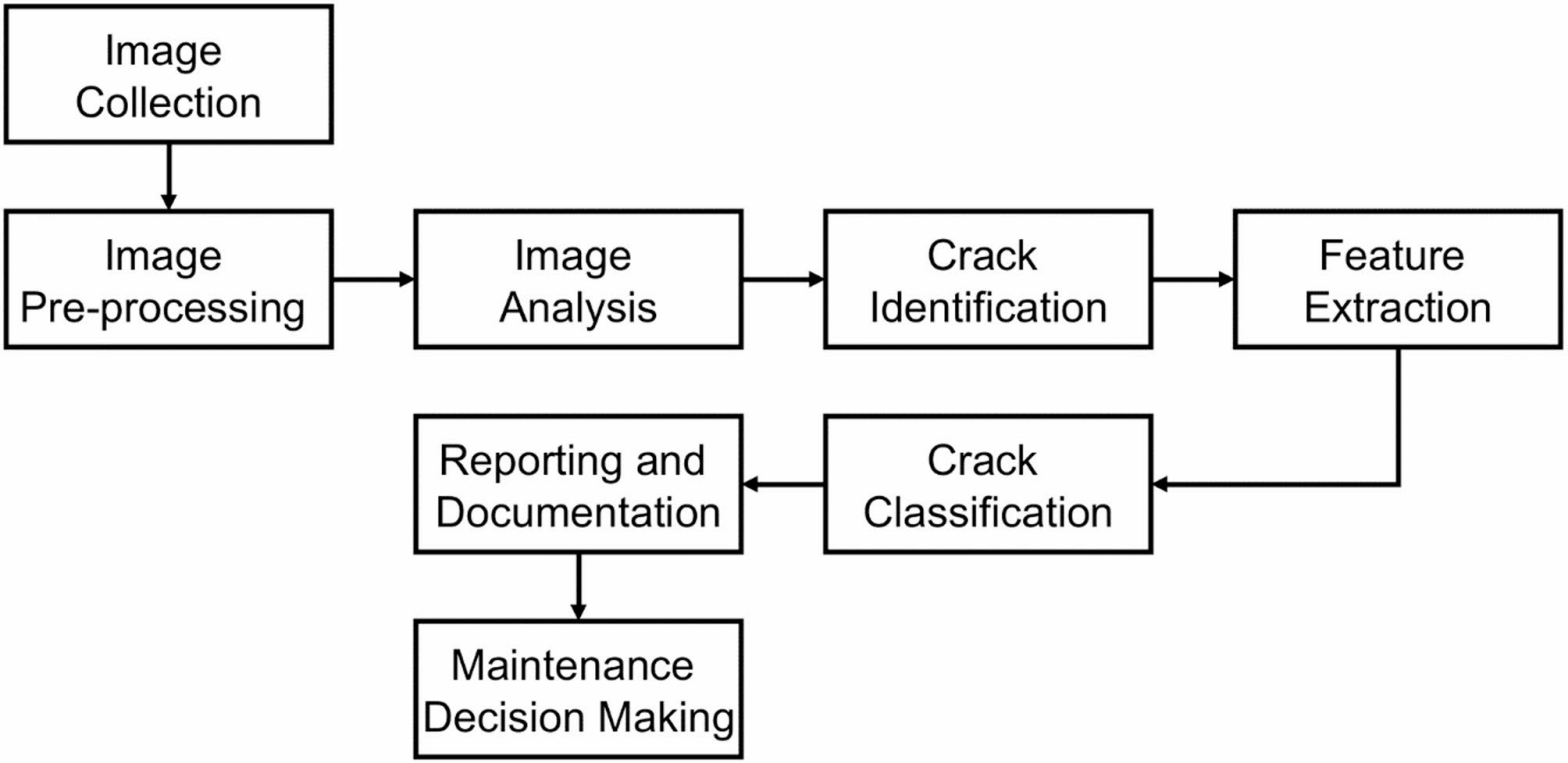
Architectural framework diagram for crack detection via image processing.

This section presents an outline of a procedure for the image processing-based detection of cracks that is both as accurate and efficient as possible. This broad framework is detailed in Fig. 2, starting from image collection for PV panels. In this phase, specialized imaging equipment is used to acquire high-resolution images of the PV panels that need inspection, guaranteeing accurate and reliable data acquisition. Subsequently, the pre-processing of collected images phase employs critical image enhancement techniques, including noise reduction, contrast correction, and segmentation, to prepare the images for an in-depth analysis. Subsequently, sophisticated image processing algorithms are implemented during the PV panel image analysis. Edge detection and pattern recognition techniques that are used on pre-processed images are a means of detecting and analyzing patterns that would indicate the presence of fractures. For the subsequent stage, the identification of cracks within PV panels, the system meticulously scrutinizes these images to accurately identify fractures by leveraging the enriched data acquired from earlier phases. While the next step in the procedure is detecting the cracks, and extracting those cracks, features such as length, breadth, depth, and propagation direction are extracted and studied. This data is critical for determining the severity of the cracks found. After features are extracted, the next step is classifying PV panel cracks according to their potential impact on the performance of the panel, thus helping in maintenance decision-making. Once the reporting and documentation phase is complete, a complete set of reports is created to give a detailed description of the characteristics and kinds of the captured fractures. Such results help maintenance teams assess the condition of panels and schedule any repairs. The final step is maintenance decision-making, which directs master maintenance decisions of whether the impacted panels need repair, maintenance, or replacement. Such decisions are backed by several extensive reports and analysis reports, ensuring operational efficiency of solar power installations over a long period of time.

## PV cell defects: impacts and research

In this section, the role of PV cell failure modes and solar PV module performance issues are explored together with the respective research. Initially, the study investigates intrinsic and extrinsic defects in PV cells and their signs. The following investigation details how a wide variety of faults, including fractures and other failures, can significantly reduce power generation—even when the damage seems minor. This cements the critical importance of accurate defect detection to maintaining efficiency. A detailed review of the relevant literature on the topic shows considerable advances in understanding and addressing these challenges. These advancements have been critical for developing better detection technology and mitigation techniques. Given the extensive nature of solar PV systems, this holistic approach adheres to the proposed framework and will highlight methodologies to amplify the efficiency, reliability, and durability of solar PV systems, alongside addressing prominent challenges and opportunities for advancement in the sector.

### Overview of PV cell defects

**Fig. 3:**
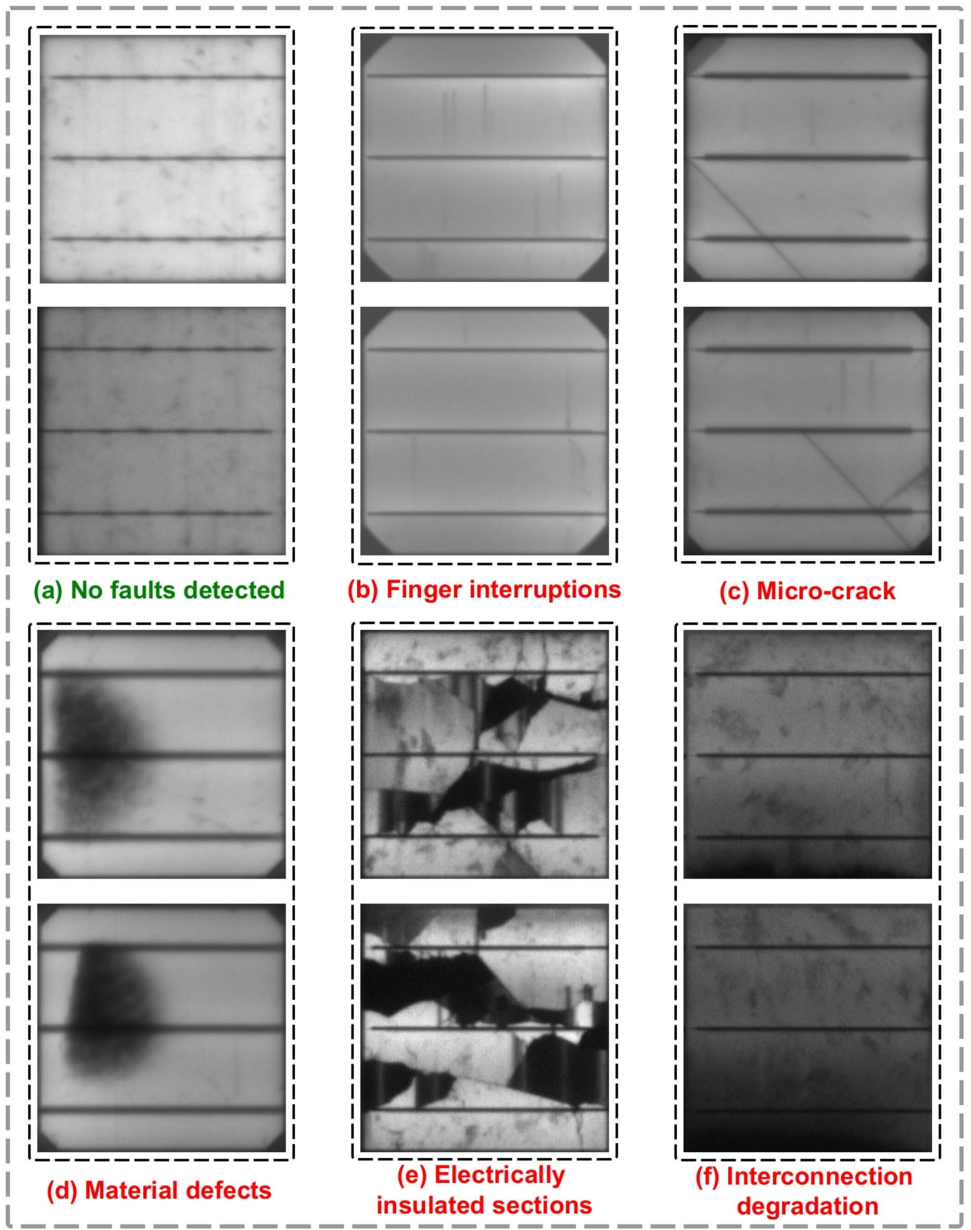
Comprehensive visualization of intrinsic and extrinsic defects.

This study proposes a Residual Network (ResNet) based image processing method for the accurate identification of fractures in PV panels, essential for enhancing their performance and durability. Figure 3 depicts many categories of defects that may arise in PV panels, including “No faults detected,” “Finger interruptions,” “Micro-crack,” “Material defects,” “Electrically insulated sections,” and “Interconnection degradation”.

PV cells are the basic units for converting sunlight into electrical energy. However, numerous defects can affect their function and life. The study imagined the ideal situation that there would be no fault in Fig. 3(a). EL imaging, a common practice in the inspection of solar modules, confirms that PV cells are performing at their design limit without sustained performance loss when in this state. It guarantees the PV system works at its optimum level^12^.

Finger interruptions seen in Fig. 3(b) arise when the conductive grid lines (fingers) inside the PV cell sustain damage or become detached. These interruptions result in heightened series resistance, which directly impacts current flow, diminishing the cell’s capacity to produce electricity. The deterioration of finger connections is mostly attributed to thermal fatigue, mechanical stress, or corrosion over time, as examined in research investigating metallization failures in crystalline silicon PV modules^13^. If untreated, this deficiency may lead to a substantial reduction in energy production due to the deterioration of electrical connections between individual cells^14^.

Micro-cracks, depicted in Fig. 3(c), are another common issue in PV cells. These little cracks in the silicon substrate often arise from mechanical strains during transit, installation, or environmental influences such as high temperature variations. Micro-cracks diminish the cell’s active area, resulting in localized power losses and hotspot development, which may expedite deterioration over time^15^. Studies indicate that these fissures may diminish the power production of PV modules by up to 0.5% more than intact modules after 21 months of operation^16^. Moreover, micro-cracks may progress into more significant fractures with time, particularly under environmental stress, resulting in increased power losses^17^.

Material defects as presented in Fig. 3(d) —these are inherent defects within the silicon wafer or other materials used to fabricate the PV cell. Manufacturing introduces defects such as impurities or structural dislocations, the presence of which can result in these above-mentioned defects. As time passes, the material deficiencies will aggravate other deficiencies, like cracking or corrosion, leading to a drop in the efficiency of the cell. Combined with environmental factors such as temperature and Ultraviolet (UV) exposure, recent literature has documented that these defects can contribute to faster degeneration of PV systems^18^. If the material defects are not found and addressed during inspections in the early stage, it will result in permanent performance losses.

Figure 3(e) illustrates electrically insulated sections that arise when parts of the cell get detached from the remainder of the module owing to significant cracking or delamination. This results in large portions of the PV module being nonfunctional, significantly reducing the total power production. Electrically insulated sections often result from Potential Induced Degradation (PID), whereby environmental conditions such as high voltage, humidity, and temperature differentials compromise the insulation of module components^18^. PID may markedly diminish the efficiency of solar panels and, if unaddressed, can incapacitate substantial areas of the module.

Finally, connector degradation is shown in Fig. 3(f), indicating the deterioration of the soldered connections among individual PV cells. This kind of deterioration often arises from mechanical fatigue caused by temperature fluctuations or inadequate soldering during production. As time progresses, the disconnection between cells escalates electrical resistance, resulting in considerable power loss. Research indicates that interconnect deterioration may lead to a loss of up to 40% in power output owing to failures in the connections between the fingers and the busbars^14^. Moreover, studies demonstrate that external environmental conditions, such as humidity and temperature fluctuations, might expedite this kind of degeneration^19^.

### Analysis of PV cell defects and their impact

Table 1 provides a comprehensive overview of the anomalies of the solar panels, like electrical performance failure, and system aging, extreme weather conditions. It provides details about particular disorders like PID, encapsulate discoloration, and hail impacts and their consequences in efficiency, structural stability, and long-term performance. Table 1 summarizes the challenges and their more general impacts on maintenance, thus identifying the urgent requirements for state-of-the-art diagnostics and predictive maintenance to ensure the reliability and sustainability of PV systems.

**Table 1:** Overview of PV cell defects, their manifestations, and implications.

Category	Description	Examples/Manifestations	Implications
Electrical performance failures	Inefficiencies caused by internal mismatches or wear.	- PID - Hotspots - Partial shading	- Efficiency losses - Overheating leading to module damage
System aging	Gradual wear of components reducing power output.	- Encapsulant discoloration - Delamination - Corrosion - Solder fatigue	- Reduced energy efficiency - Compromised structural integrity
Extreme weather conditions	Environmental stress accelerating damage.	- Hail impacts - UV degradation - Snow accumulation	- Misalignment or damage - Decreased lifespan and reliability
Broader maintenance implications	Need for advanced diagnostics and proactive strategies.	- Thermal imaging for hotspots - EL imaging	- Improved reliability and sustained performance

### Dataset imbalance strategies

**Fig. 4:**
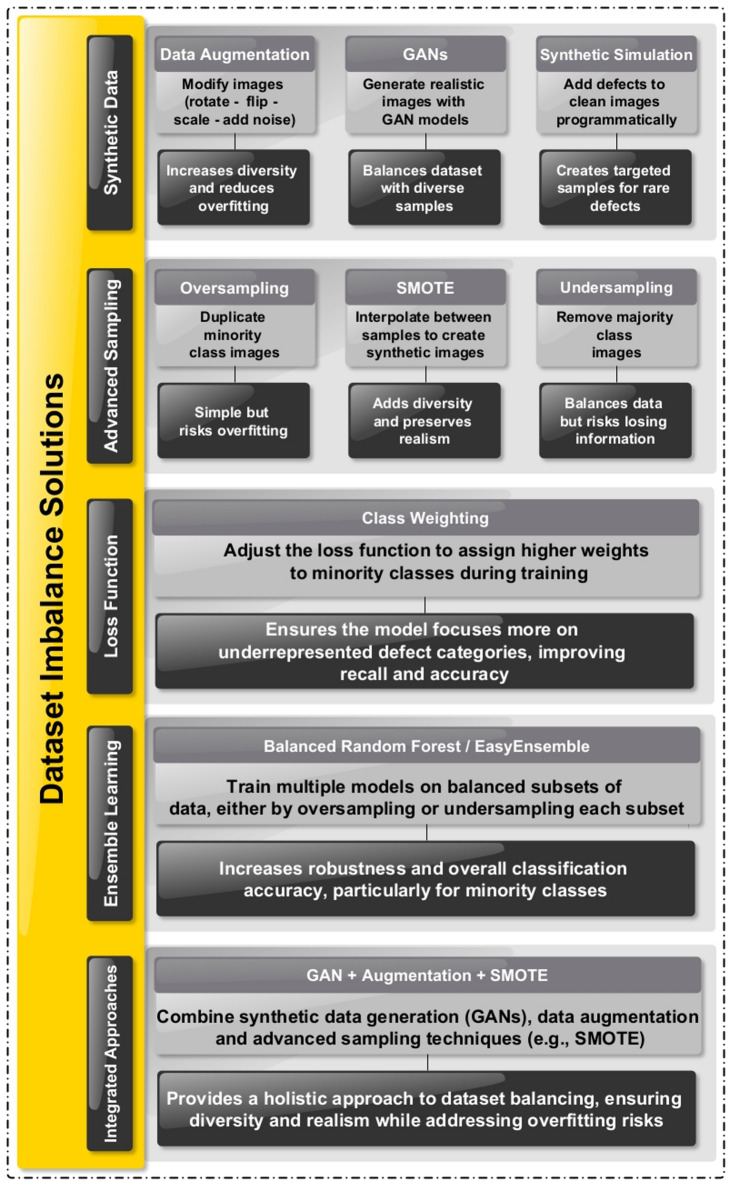
Methods for addressing dataset imbalance in Image-Based classification.

Figure 4 delineates many ways for mitigating dataset imbalance in image categorization. The approaches include synthetic data generation techniques, such as data augmentation, Generative Adversarial Networks (GANs), and synthetic simulation, as well as advanced sampling techniques including oversampling, Synthetic Minority Oversampling Technique (SMOTE), and undersampling, each providing distinct advantages for improving data diversity and balance. Furthermore, loss function adjustment aims to promote minority class recognition by assigning weights to misclassifications, whereas ensemble learning uses balanced subsets to improve model resilience and accuracy. Ultimately, Integrated approaches amalgamate many strategies such as GANs, augmentation, and SMOTE to deliver a thorough and efficient remedy for dataset imbalance. This figure functions as a reference for enhancing model performance in situations with underrepresented groups.

### Effects of cracks on PV module efficiency

**Fig. 5:**
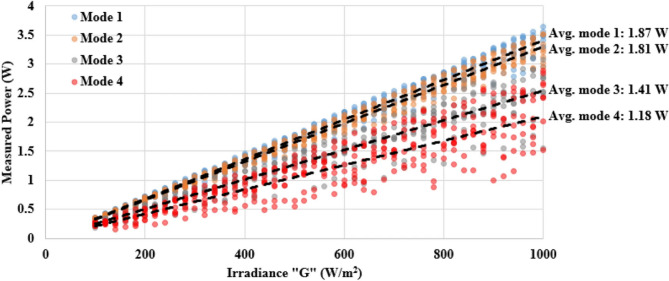
Measured power vs. irradiance of the examined solar cells ‎^20^.

The study ‎^20^ conducts a current inquiry to analyze the effects of different fracture sizes on solar cells. Cracks were divided into four categories. Mode number one cells has no cracks. Mode number two has micro-cracks, whereas mode number three has shaded cracks. The fourth stage entails cellular disintegration. Figure 5 illustrates that increased cracks in solar cells result in around 60% of power loss. Elevated temperatures diminish PV module efficiency and may decrease power output. The SolarQRNN model ‎^5^ utilizes Deep Learning (DL) methods, using a distinctive quintile loss function and backbone networks comprised of residual convolution units. The model exemplifies a novel methodology for using tracking camera images to assess the likelihood of solar power loss due to soiling.

### Related studies

Table 2 presents a systematic overview of significant research in the domain of PV panel failure detection. It offers a comprehensive summary of the accomplishments and methodologies in this field, including details on the references, year, used methods, study descriptions, and significant findings.

**Table 2:** Overview of related studies in PV panel defect detection research.

Reference	Year	Technique Used	Study Description	Major Findings
^21^	2024	Knowledge distillation with YOLOX and CSPHN networks.	Employs a bi-branch collaborative training method with knowledge distillation for PV hot-spot identification, emphasizing improvements in detection precision and computational efficiency.	Attained an AP50 metric of 82.2%, indicating rapid and precise hot-spot defect identification over diverse adverse situations.
^22^	2024	DL using surveillance camera images.	Created SoilingEdge, a DL methodology for estimating power loss from the soiling of PV panels from images obtained from edge devices such as security cameras.	Accomplished precise power loss assessment from soiling by advanced image processing techniques on edge platforms, enabling reliable inference for outdoor PV monitoring.
^23^	2023	Ghost Convolution Including YOLOv5 with BottleneckCSP and Tiny Target Prediction Head (GBH-YOLOv5)	The study introduces GBH-YOLOv5, a new method for detecting tiny defects on PV panels using advanced image processing.	Achieving at least a 27.8% increase in accuracy compared to state-of-the-art methods.
^24^	2022	End-to-end approach using Red Green Blue (RGB) imagery from Unmanned Aerial Vehicles (UAVs) and YOLOv4 architecture for DL-based defect detection.	The study develops a method using UAV-acquired RGB imagery and YOLOv4 to detect, identify, and locate defects in solar PV modules.	The defect detection achieved 83% accuracy on the validation set and 73% on the test set in large-scale PV installations.
^25^	2022	DL using ResNet152-Xception and a coordinate attention mechanism.	Deep-learning model using ResNet152-Xception and attention for PV cell defect detection, addressing data scarcity and imbalance.	The model achieves 96.17% accuracy in binary classification and 92.13% in multiclassification of PV cell defects, outperforming several common models.
^26^	2021	U-net semantic segmentation for EL image analysis of PV modules.	The study utilizes U-net architecture to detect and quantify defects in solar PV modules through EL imaging, spanning various module designs and image qualities.	Defects in silicon wafer-based solar cells that are mono- or multi-crystalline are efficiently identified and measured by the U-net model.
^27^	2021	Synchronized Thermography (ST) using a portable IR-camera.	The research investigates PV fault identification by Infrared Radiation (IR) thermography, emphasizing the adaptation of observations to varying outside situations.	IR thermography is effective in detecting PV panel defects in various harsh conditions, providing consistent information with common conditions.
^28^	2021	Optical Stepped Thermography with post-data processing algorithms.	The study utilizes optical stepped thermography, halogen lamps, and IR camera monitoring to enhance defect signatures in PV panels.	This method effectively identifies defects in PV panels, with processed images being more evaluative than raw thermal images.
^29^	2020	Support Vector Machine (SVM) and Back Propagation Neural Network (BPNN)	identification of solar cell microcracks by EL image analysis.	Achieved 92.67% accuracy with SVM and 93.67% with BPNN in classifying solar cells as cracked or not.
^30^	2019	Steerable evidence filtering, local thresholding, and a minimum spanning tree for crack detection.	Identifying fissures in multicrystalline solar cells with improved contrast crack saliency maps and segmentation techniques for comprehensive crack extraction.	94.4% average detection rate for various types of cracks.
^31^	2019	Machine learning (ML) - SVM, Random Forest (RF) with Hough transform for pattern detection.	Automatic splitting of EL images into cells, defect detection, and feature computation for precise defect categorization in PV panels.	Achieved high accuracy (0.997) but lower recall (0.274) using SVM in defect type identification.

Recent years have seen the evolution of Computer Vision (CV) and its successful application across various domains. Medical informatics has employed CV-based DL models, particularly for monocular depth estimation from fundus photographs, resulting in impressively accurate early diagnosis and monitoring of ophthalmic diseases^32^. For instance, the construction industry has utilized 3D vision technology to construct brick-and-mortar structural crack damage recognition robots, automating the accurate and efficient detection of external cracks in buildings, thereby enhancing safety and lowering construction and maintenance costs^33^. Such applications demonstrate the capacity of CV to analyze intricate visual data, derive valuable information, and assist in crucial decision-making processes. A recent research examined superficial problem detection in solar panels, addressing concerns like dust and bird droppings, employing a Convolutional Neural Network (CNN) based VGG16 model in conjunction with a PyQt5 interface for intuitive categorization. The model attained an F1 score of 91.67%, specificity of 98.29%, and accuracy of 91.46% using a dataset of six fault classes with enhanced images^34^. Based on these results, our study shows that CV methods, specifically ResNet-based DL architectures, could be used to solve the important problem of finding faults in PV panels. The ultimate goal is to make it easier to maintain clean energy.

### Advancing PV inspection: method comparison for EL defect detection

Table 3 summarizes the proposed ResNet34-based method, which achieves robust generalization and fine-grained fracture classification (micro/macro) and addresses critical limitations of traditional ML and conventional DL approaches. It optimizes for peripheral deployment through Open Neural Network Exchange (ONNX), utilizing residual learning and class-balancing strategies to achieve competitive performance. Even in the presence of variable image quality, this framework surpasses previous methods in terms of adaptability and scalability in real-world photovoltaic inspection systems.

**Table 3:** Comparative analysis of ML approaches for photovoltaic defect detection in EL imagery: traditional methods, conventional DL, and ResNet34-Based optimization.

Aspect	Traditional ML	Conventional DL	Proposed Method
Input type	Handcrafted features	Raw EL/IR/RGB images	Raw EL images (high resolution)
Feature extraction	Manual engineering	Automatic (convolutional layers)	Automatic via residual blocks with deep feature representation
Model complexity	Low to moderate	Moderate to high	Moderate (ResNet34 strikes balance)
Accuracy	Low to moderate	High	High
Training data requirements	Small to medium datasets	Large datasets	Optimized for medium-sized dataset with class balancing
Generalization	Limited (noise-sensitive)	High (but overfits on imbalance)	High; robust across defect types due to residual learning and augmentation
Computation efficiency	Very efficient	Varies; heavier models (e.g., YOLOv5, ResNet152) require high compute	Efficient; suitable for edge deployment with ONNX export
Detection types	Binary	Binary or multiclass (limited microcrack sensitivity)	Fine-grained (micro/macro, dormant)
Deployment readiness	Offline tools	Resource-intensive	Edge devices (low power)
Robustness to image quality	Low (needs clean input)	Moderate	High due to preprocessing + skip connections in ResNet
Scalability to real-world PV systems	Low	Moderate to high	High (modular, portable)

### Novel contributions and practical impact

**Fig. 6:**
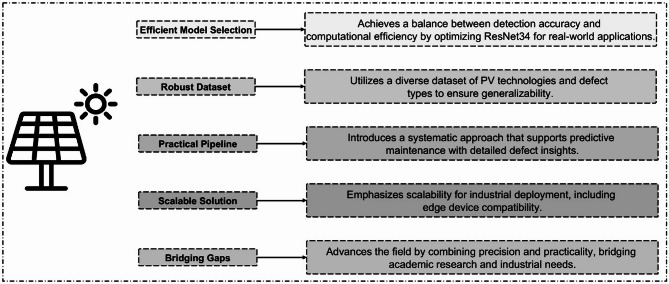
Key novel contributions of the proposed PV defect detection approach.

The study’s novelty, as shown in Fig. 6, lies in its balanced approach, optimizing ResNet34 for efficient and accurate PV defect detection. A robust dataset ensures generalizability, while the systematic pipeline supports predictive maintenance. The scalable solution bridges the gap between academic research and industrial applications, enabling deployment on edge devices.

## Methodology

In the methodology section, the study carefully examines the key aspects of the approach to detecting cracks in PV panels. Section 3.1 specifically examines the use of ResNet in the detection algorithm. Section 3.2 delineates the systematic procedural methodology the study utilized. Section 3.3 explores the training, assessment, and prediction procedures of the DL model. The dataset that is crucial to the investigation is analyzed in Sect. 3.4, while Sect. 3.5 is specifically focused on the mathematical techniques employed. Every component is crucial in showcasing the thorough methodology for identifying and evaluating faults in PV panels.

### ResNet in PV crack detection

The research uses the ResNet architecture among several backbone networks. Its layers include skip/shortcut connections, which marked a significant breakthrough in the training of deep networks.

**Fig. 7:**
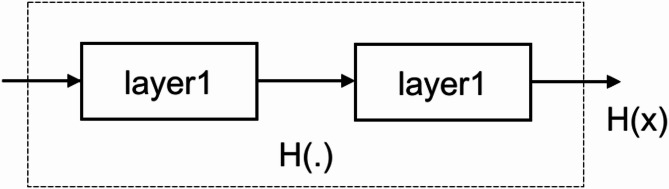
Illustration of gradient vanishing in DL architectures^35^.

DL networks, particularly those employed in CV applications, require a substantial number of layers to attain sufficient capacity. Nevertheless, the inclusion of more layers might provide greater difficulty in training as a result of the vanishing gradient problem. The issue is emphasized in Fig. 7, illustrating a section of the network $\:H\left(\text{x}\right)$ with layer 1 and layer 2. When the partial derivative of $\:H\left(\text{x}\right)$ with respect to x is noticeably smaller than 1, the vanishing gradient issue arises, which is a typical issue when using activation functions like Sigmoid. This produces a significant drop in the gradient of $\:\text{x}$, as (1) explains^35^. Deep network training is seriously challenged by the well-known phenomena of the gradient vanishing issue. ResNet effectively addresses this problem by using a creative design, therefore optimizing the training process.

**Fig. 8:**
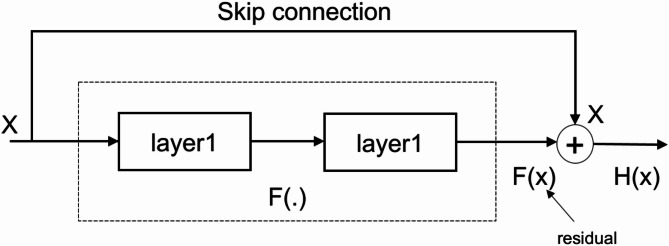
Illustration of skip connections within ResNet architecture^35^.

Nevertheless, adding a shortcut or skip link from $\:\text{x}$ to $\:H\left(\text{x}\right)$, as seen in Fig. 8, streamlines the learning process. In this setup, it is the network’s responsibility to learn about the residue, $\:F\left(\text{x}\right)=H\left(\text{x}\right)-\text{x}$ which is shown to be a more feasible and controllable method. This expression is called (2), and by feeding this skip connection The gradient of $\:\text{x}$ propagates in a more direct manner, directly from the gradient of $\:H\left(\text{x}\right)$. In short, this adjustment minimizes the effect of vanishing gradient problem. Such that, the task for which the study needs to learn $\:H\left(\text{x}\right)$ is even more difficult as per (3). The use of a skip connection allows you to carry information about x from the network, making learning even faster^35^.

1 $$\:\left|\frac{\partial\:H\left(\text{x}\right)}{\partial\:\text{x}}\right|\ll\:1$$

2 $$\:F\left(\text{x}\right)=H\left(\text{x}\right)-\text{x}$$

3 $$\:\left|\frac{\partial\:H\left(\text{x}\right)}{\partial\:\text{x}}\right|=\left|\frac{\partial\:F\left(\text{x}\right)+\partial\:\text{x}}{\partial\:\text{x}}\right|=\left|\frac{\partial\:F\left(\text{x}\right)}{\partial\:\text{x}}\right|+1$$

**Fig. 9:**
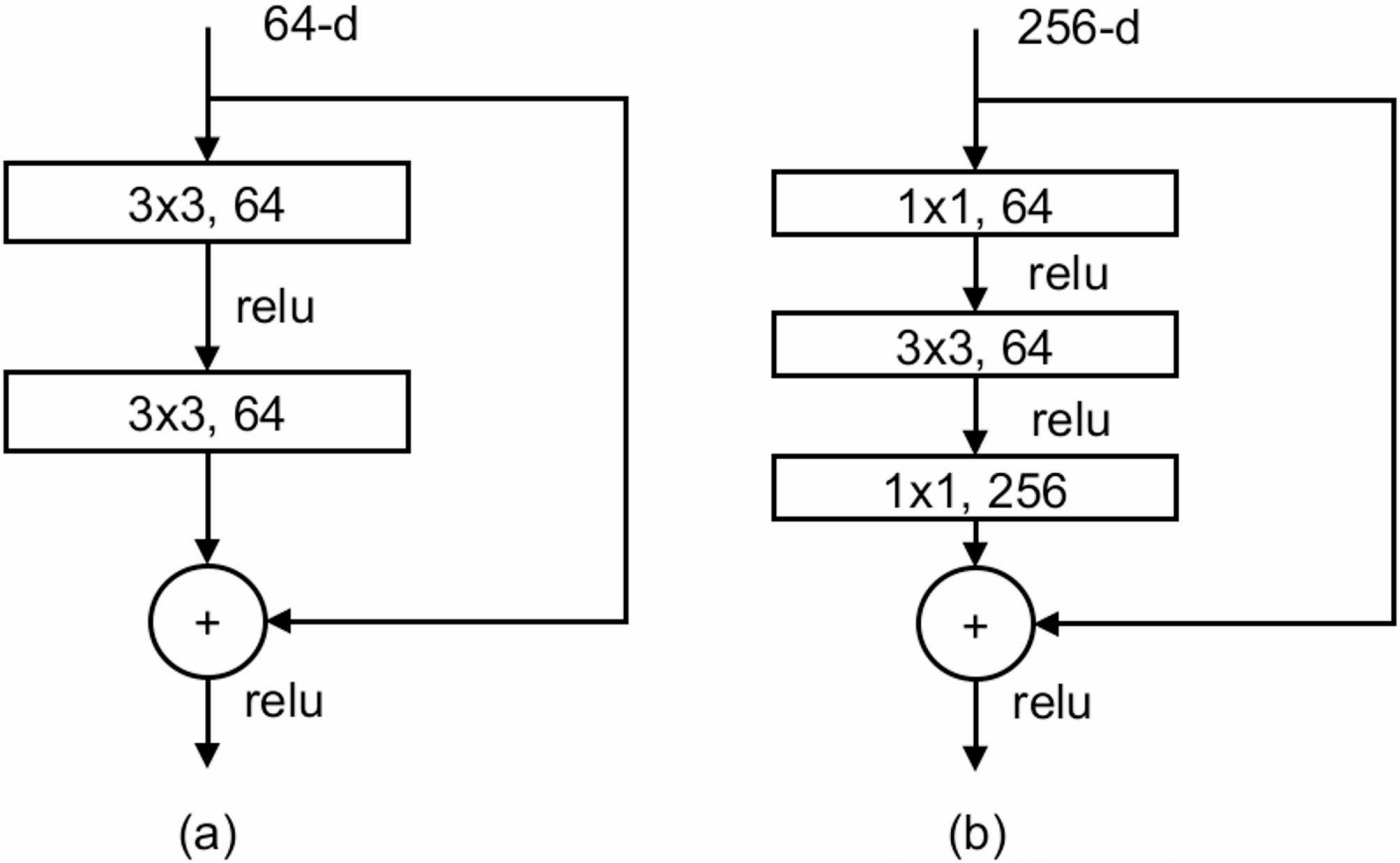
Comparison of ResNet residual blocks: (a) ResNet 18 and 34 standard residual blocks vs. (b) Advanced residual blocks in ResNet 50, 101, and 152^35^.

In Fig. 9 the contrast in the residual block designs may be observed between ResNet-18 and ResNet-34, as well as between ResNet-50, ResNet-101, and ResNet-152. The ResNet-50 architecture utilizes a bottleneck design, which has a combination of 1 × 1 and 3 × 3 convolutional layers in an alternating pattern^35^. This strategy significantly reduces the number of parameters in the model, dropping them from 294,912 (as in a configuration with two 3 × 3 convolutions of 64 and 3 × 3, 256) to 69,632. Adding this bottleneck framework also increases the number of activations because it has more convolutional layers. This makes the model more nonlinear and increases its overall capacity.

As Table 4 shows, the ResNet family—which comprises ResNet 18, ResNet-34, ResNet-50, ResNet-101, and ResNet-152—is distinguished by its unique arrangement of residual blocks and their numbers. Five major convolutional layers (conv1 through conv5_x) make up the ResNet model; then it has an average pooling layer, a fully connected layer, and a softmax layer. For the ImageNet dataset, this mix essentially serves as the classifier for 1000 different object types. Often referred to as the “backbone” of the ResNet models, the convolutional layers initially learn on ImageNet to gather information at varying degrees of detail. Features maps following conv2_x, conv3_x, conv4_x, and conv5_x clearly show this. Using exact neck and head configurations to complete such like item recognition and semantic segmentation alters these features even more. The essential component of these ResNet designs is the residual block, which is employed in different quantities across the convolutional layers, ranging from conv2_x to conv5_x. Whereas ResNet-50 uses a unique mix of 3, 4, 6, and 3 blocks in these layers, respectively, ResNet-18 uses 2 residual blocks in each layer from conv2_x to conv5_x.

**Table 4:** ResNet architectures: Layer-By-Layer specifications^35^.

layer name	output size	18-layer	34-layer	50-layer	101-layer	152-layer
conv1	$\:112\times\:112$	7 × 7,64 stride 2				
conv2_x	$\:56\times\:56$	3 × 3 max pool, stride 2				
		$$\:\left[\begin{array}{c}3\times\:3,\:64\\\:3\times\:3,\:64\end{array}\right]\times\:2$$	$$\:\left[\begin{array}{c}3\times\:3,\:64\\\:3\times\:3,\:64\end{array}\right]\times\:3$$	$$\:\left[\begin{array}{c}1\times\:1,\:64\\\:3\times\:3,\:64\\\:1\times\:1,\:256\end{array}\right]\times\:3$$	$$\:\left[\begin{array}{c}1\times\:1,\:64\\\:3\times\:3,\:64\\\:1\times\:1,\:256\end{array}\right]\times\:3$$	$$\:\left[\begin{array}{c}1\times\:1,\:64\\\:3\times\:3,\:64\\\:1\times\:1,\:256\end{array}\right]\times\:3$$
conv3_x	$\:28\times\:28$	$$\:\left[\begin{array}{c}3\times\:3,\:128\\\:3\times\:3,\:128\end{array}\right]\times\:2$$	$$\:\left[\begin{array}{c}3\times\:3,\:128\\\:3\times\:3,\:128\end{array}\right]\times\:4$$	$$\:\left[\begin{array}{c}1\times\:1,\:128\\\:3\times\:3,\:128\\\:1\times\:1,\:512\end{array}\right]\times\:4$$	$$\:\left[\begin{array}{c}1\times\:1,\:128\\\:3\times\:3,\:128\\\:1\times\:1,\:512\end{array}\right]\times\:4$$	$$\:\left[\begin{array}{c}1\times\:1,\:128\\\:3\times\:3,\:128\\\:1\times\:1,\:512\end{array}\right]\times\:8$$
conv4_x	$\:14\times\:14$	$$\:\left[\begin{array}{c}3\times\:3,\:256\\\:3\times\:3,\:256\end{array}\right]\times\:2$$	$$\:\left[\begin{array}{c}3\times\:3,\:256\\\:3\times\:3,\:256\end{array}\right]\times\:6$$	$$\:\left[\begin{array}{c}1\times\:1,\:256\\\:3\times\:3,\:256\\\:1\times\:1,\:1024\end{array}\right]\times\:6$$	$$\:\left[\begin{array}{c}1\times\:1,\:256\\\:3\times\:3,\:256\\\:1\times\:1,\:1024\end{array}\right]\times\:23$$	$$\:\left[\begin{array}{c}1\times\:1,\:256\\\:3\times\:3,\:256\\\:1\times\:1,\:1024\end{array}\right]\times\:36$$
conv5_x	$\:7\times\:7$	$$\:\left[\begin{array}{c}3\times\:3,\:512\\\:3\times\:3,\:512\end{array}\right]\times\:2$$	$$\:\left[\begin{array}{c}3\times\:3,\:512\\\:3\times\:3,\:512\end{array}\right]\times\:3$$	$$\:\left[\begin{array}{c}1\times\:1,\:512\\\:3\times\:3,\:512\\\:1\times\:1,\:2048\end{array}\right]\times\:3$$	$$\:\left[\begin{array}{c}1\times\:1,\:512\\\:3\times\:3,\:512\\\:1\times\:1,\:2048\end{array}\right]\times\:3$$	$$\:\left[\begin{array}{c}1\times\:1,\:512\\\:3\times\:3,\:512\\\:1\times\:1,\:2048\end{array}\right]\times\:3$$
	$\:1\times\:1$	Average pool, 1000-d fc, softmax (classification head)				
FLOPs (Floating Point Operations per Second)		$\:1.8\times\:{10}^{9}$	$\:3.6\times\:{10}^{9}$	$\:3.8\times\:{10}^{9}$	$\:7.6\times\:{10}^{9}$	$\:11.3\times\:{10}^{9}$

### Procedural workflow of PV panel crack detection

The study proposes an advanced DL system that actuated accurately the fracture detection and analysis of the PV container Designed in Python and built on “PyTorch”, one of the most popular DL tools, this system approach is primarily sustained on the “PVPanelCrackDetector” class, liable for managing the whole training and model verification process. The study adopts a modified ResNet architecture which is already recognized for its performance in image related tasks. This framework can be initialized with a pre-existing state or trained from scratch.

The model may be trained with the help of parameters such as the number of epochs, learning rate, batch size, and validation split ratio. Thus allowing for highly specific customization based on dataset characteristics and flexibility. Data loading and effective management of training and validation data sets are handled by the “DataLoader” from PyTorch. A significant property of the approach is the ability to resume training from a checkpoint, thus improving the usability of active research studies.

The evaluation and deployment procedure is based on the “main_test”. It loads acquired models and forecasts cracks on fresh PV panel images. This feature also allows one to export the trained model to the ONNX format, a portable model file crucial for use in many production environments. Furthermore, the study efficiently manages experimental setups using a utility function known as “experiment_deleter,” which helps us to readily eliminate tests and iterate rapidly throughout the development period.

A full range of the major configurations and parameters used in the image processing system is given in Table 5. The approach results show a promising performance in locating the cracks in PV panels. It not only improves the quality of service within the sustainable energy sector by ensuring solar panel functionality but also sets a foundation where advanced image processing techniques are applied within renewable energy solutions, further improving operational metrics. Its modular structure and holistic perspective pave the way for further research in the area of image-based anomaly detection in solar systems.

**Table 5:** Configuring ResNet for PV panel crack detection.

Parameter	Quantity
Optimizer	Adam
Loss function	BCEWithLogitsLoss
Learning rate	7e-5
Batch size	32
Validation split ratio	0.2
Resume training	Configurable (True/False)
Model architecture	ResNet
Data loader workers	4
Training data handling	DataLoader (PyTorch)
Stopping patience	10

**Fig. 10:**
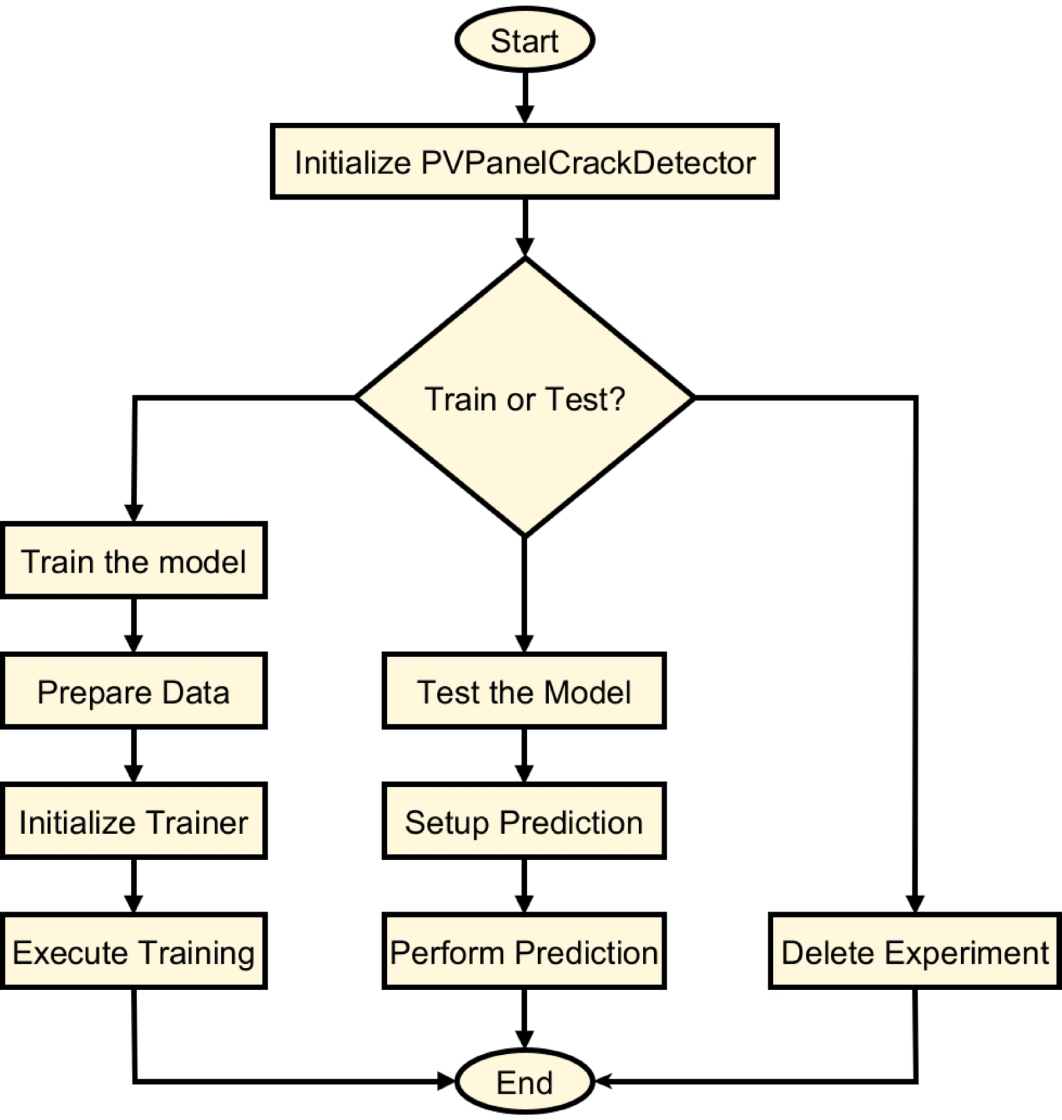
Flowchart for ResNet-Driven PV panel crack detection.

Figure 10 offers a methodical and orderly graphical depiction of the same approach used in the research. The “PVPanelCrackDetector” class’s initial setup starts the flowchart and is the basis for the ResNet-based study. It then veers into three distinct operational directions: training, testing, and experiment cancellation. The model completes many fundamental tasks throughout the training process, including data preparation, trainer setup, and execution. Accurately spotting problems in solar panels depends on these processes. Showing the useful use of the trained model, the testing path comprises the setup for forecasting and the subsequent assessment of prediction accuracy. At last, the deletion route consists of the maintenance element of the process, allowing the deliberate removal of experiments based on certain criteria. The flowchart not only improves understanding of the approach but also emphasizes the flexibility and completeness of the system in addressing the problems related to spotting solar panel fractures.

### DL model development and assessment

The study had resulted in a comprehensive Python framework that integrates multiple computational frameworks with specific modules. This architecture is critical for the application of DL algorithms to identify defects in solar panels. The opening part of the screenplay is the importing of the needed libraries that are used in the script, for example, the “time” library used for keeping track of the time and the “os” library used for interacting with the operating system. Crucially important for the DL component of the study is “PyTorch”, often referred to as “torch.” The study also makes use of “torchvision,” a tool with CV features and datasets. Furthermore, included in the script are user-defined modules from the “configs” and “utils” folders: “serde” for the serialized format of configuring and vice versa, and “stopping” for early halting callbacks. This highlights the modular and flexible nature of the script. The “Training” class, which manages the whole training process—including validation stages—is the center of the script. The class is started with given settings, including the location of the configuration file, the stopping patience, and the epoch count. This emphasizes the capacity for personalizing the learning experience. Establishing the Compute Unified Device Architecture (CUDA) environment to use the Graphics Processing Unit’s (GPU’s) capabilities, building up the neural network model with its optimizer and loss function, and managing checkpoint loading to enable continuous training from a saved state define the major characteristics of this class. The ‘train_epoch’ and ‘valid_epoch’ approaches respectively exactly indicate the training and validation procedures for each epoch, therefore assuring a methodical and iterative process of learning and evaluation. The ‘Prediction’ class is explicitly intended for the post-training phase, where it manages the prediction or testing process by using the acquired model. This course highlights the model’s deployment capabilities, including the configuration of the CUDA environment for prediction tasks and the capacity to save the model in the ONNX format, facilitating deployment across multiple environments.

The script includes utility functions and constants such as the ‘load_pretrained_model’ function, which demonstrates the use of a pre-trained ResNet model that has been adapted for the specific goal of detecting cracks in solar panels. This function demonstrates the script’s capacity to adapt to several neural network designs that are relevant to image processing jobs.

The training procedure is quite advanced, encompassing the initial configuration of the model and optimizer, loading of data using “DataLoaders”, and executing training across numerous epochs. Every epoch consists of a training phase to teach the model and a validation phase to assess its performance on a different dataset.

In order to improve the effectiveness of training and prevent overfitting, the script includes early stopping mechanisms and the ability to store and load model checkpoints. This not only enhances the efficiency of the training process but also guarantees the dependability and resilience of the taught model. In addition, the use of “TensorBoardX” enables a user-friendly and graphical depiction of the training procedure, facilitating the observation and examination of crucial metrics such as loss and accuracy.

The Python script the study has built is a crucial part of the study. It offers a strong and adaptable framework for applying DL methods to identify faults in solar panels. The script’s architecture, which includes state-of-the-art computational libraries and customized modules, demonstrates its ability to effectively handle and analyze intricate image data. This eventually makes a substantial contribution to the progress of solar panel inspection procedures.

**Fig. 11:**
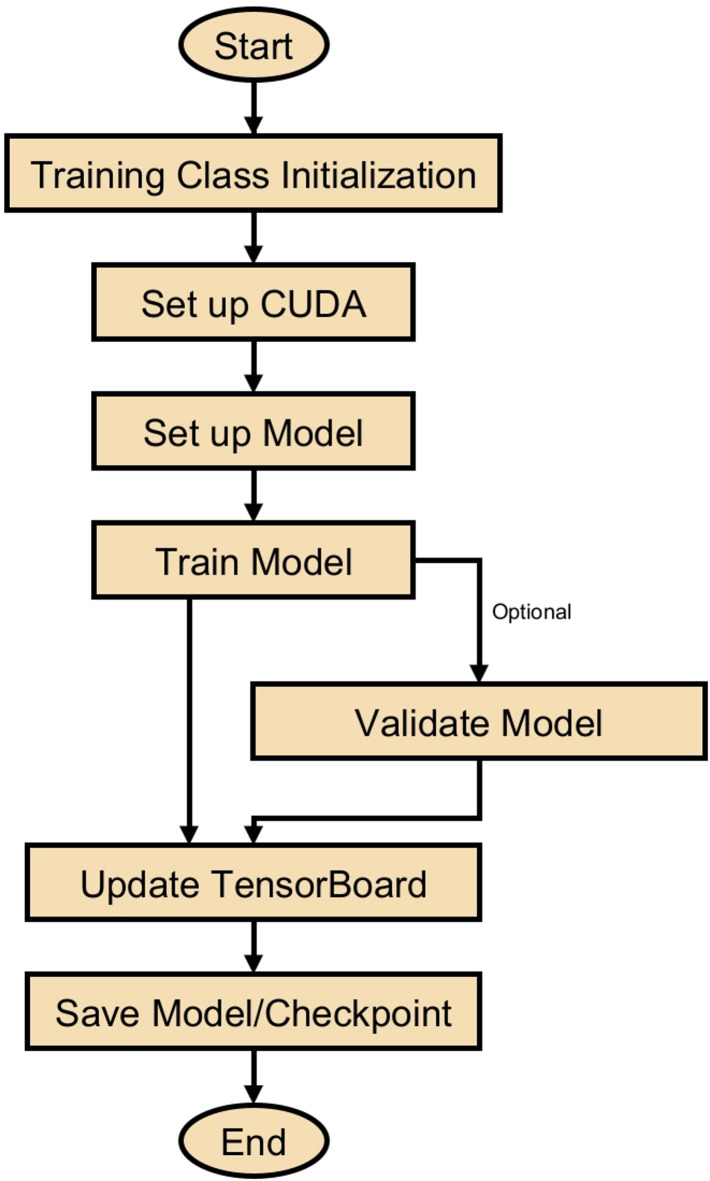
Training process flowchart for DL-based PV panel crack detection.

Figure 11 offers a thorough and unambiguous graphic depiction of the sequential processes used in training the DL model to detect solar panel cracks. Initializing the training class initiates the process, where this study develops the model’s configuration and prepare the CUDA environment for effective computation. This study has carefully developed the model to incorporate the optimizer, neural network architecture, and loss function configuration tailored to the specific requirements. The training phase, in which the model goes through rigorous training cycles with optional validation steps to ensure robustness and accuracy, is the main emphasis of the flowchart. This process depends critically on continuous monitoring and recording of training metrics in “TensorBoard”, which provides important information about the performance of the model and helps to enable exact changes. Storing the model and its checkpoints marks the end of the training period and the process. The flowchart not only helps us understand the training strategies of the model but also highlights the meticulous and thorough approach the study has used in developing a reliable tool for spotting solar panel flaws.

**Fig. 12:**
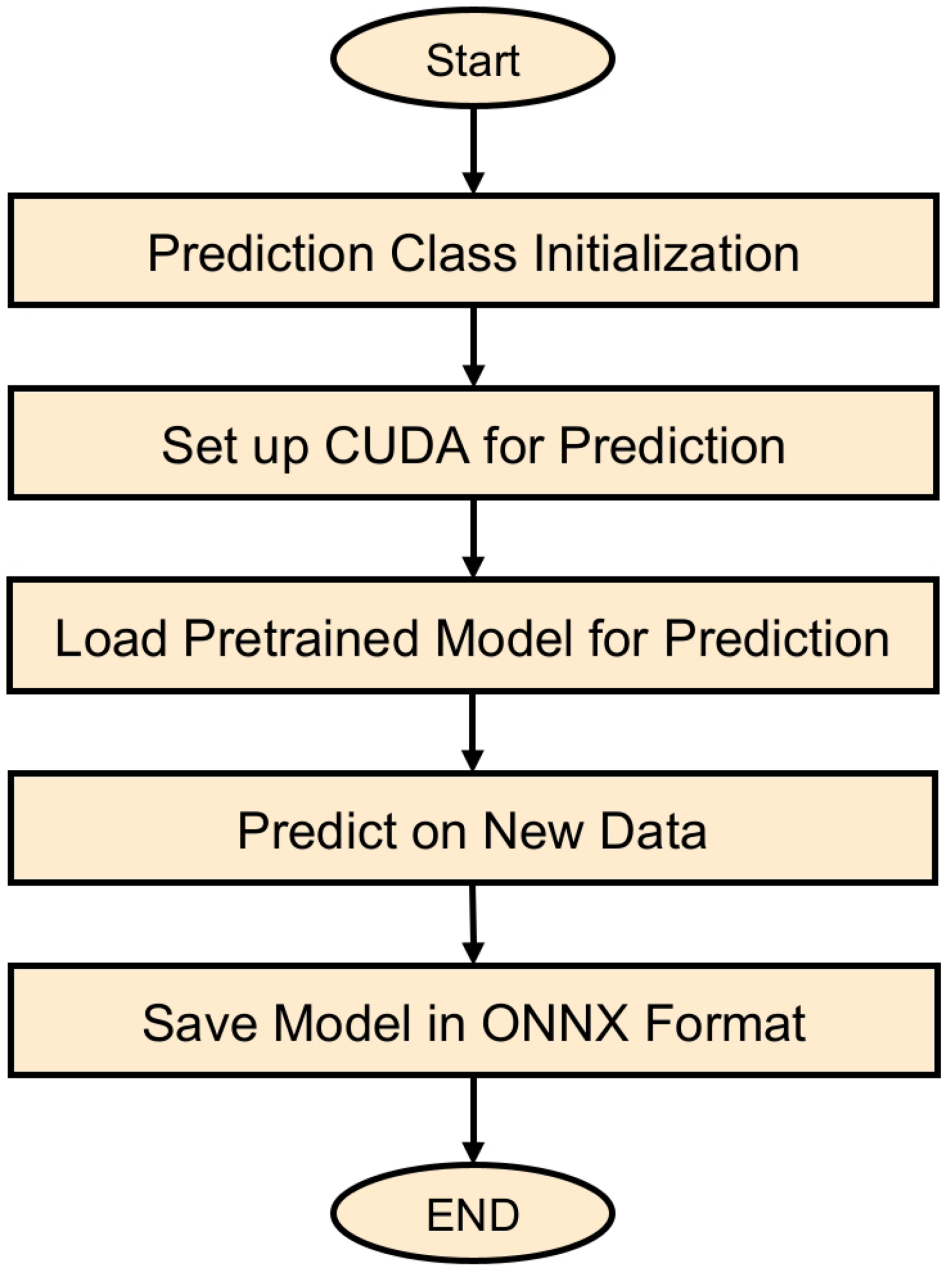
Prediction flowchart for DL-based crack detection in PV panels.

The proposed comprehensive methodology to predict and analyze fractures in PV panel fault analysis using advanced DL model is depicted in flowchart shown in Fig. 12. The first stage of the workflow is the instantiation of the prediction class itself, during which a system configuration is read and setup, ensuring that all parameters are set correctly with respect to the model. Afterwards, the flowchart illustrates the essential process of establishing the CUDA environment, which guarantees the highest level of computing performance for the prediction jobs. Subsequently, the workflow illustrates the process of loading the pre-trained model, which has been customized expressly for the purpose of detecting cracks in solar panels. While the flowchart does not clearly specify this phase, it is assumed that it is followed by the real prediction process on fresh data, which is a crucial aspect of the model’s implementation. The last phase of the workflow is preserving the model in the ONNX format, which not only signifies the completion of the prediction process but also streamlines the model’s deployment and future use. This flowchart provides a concise and organized visual depiction of the prediction process, emphasizing the methodical approach used to use DL for the efficient and precise identification of cracks in solar panels.

### Dataset presentation

The cross-domain study is based on a large dataset from the PV power-generating, specifically focusing on EL images of PV cells. The datasets used in this study are publicly available^36–38^, but were reduced to 2000 of the 2624 images, as shown in Table 6. The dataset presented a diverse variety of PV modules, including 44 modules in total, of which 18 were monocrystalline and 26 were polycrystalline. Each image in the dataset shows a PV cell with varying types of faults. To ensure consistency and simplify review, all images were resized to 8-bit grayscale images of 300 × 300 pixels while normalizing for size and view angle.

**Fig. 13:**
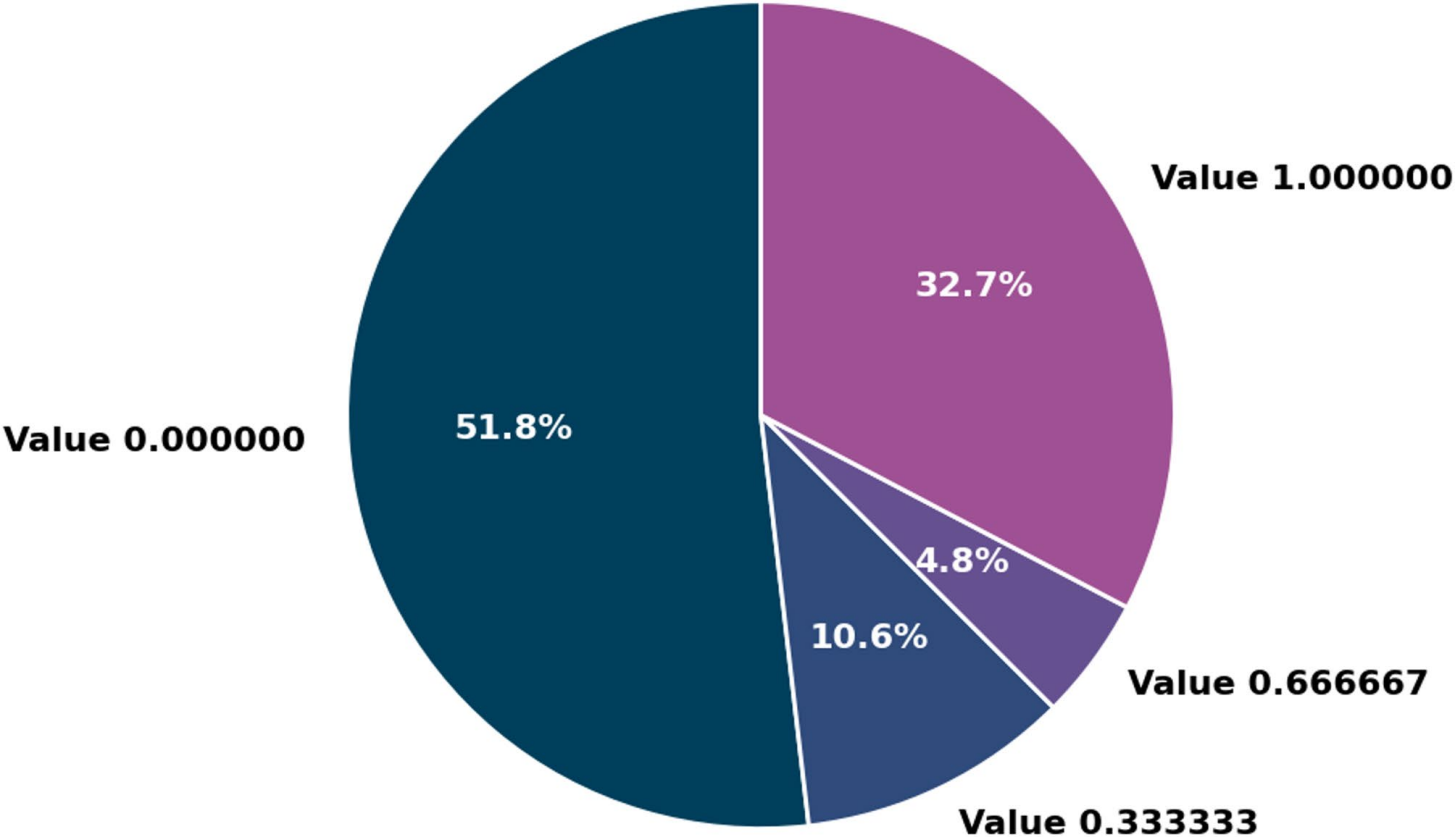
Distribution of defect probabilities in PV cell images.

A crucial aspect of the research involves using pre-categorized PV cell images, as described in Table 7; Fig. 13. The specialists carefully categorized the images into four unique groups, each reflecting a different level of chance of defects: 0% (1037 occurrences), 33% (213 occurrences), 67% (96 occurrences), and 100% (654 occurrences). The categorization offered a detailed comprehension of the range of defects present in the PV cells.

In addition, the study deliberately partitioned the dataset into three subsets, as illustrated in Table 8, for the specific objectives of training, validation, and testing. The training set, which makes up 70% of the entire dataset, consisted of 725 undamaged images (36.25%) and 675 damaged images (33.75%). The validation set consisted of 20% of the dataset, which was divided into 207 intact images (10.35%) and 193 defective images (9.65%). The testing set comprised the remaining 10% of the data, consisting of 103 undamaged images (5.15%) and 97 defective images (4.85%). The division was crucial for the strength of the investigation, guaranteeing a thorough assessment of the model’s performance in various circumstances and the possibility of defects inside the PV cells, also the dataset preprocessing and annotation details are summarized in Table 9.

**Table 6:** Dataset composition.

Type of Cells	Polycrystalline	Monocrystalline	Total
Non-defected	538	499	1037
Defected	502	461	963
Total	1040	960	2000

**Table 7:** Distribution of defect probabilities in PV cell images.

Value	Occurrences
0.000000	1037
0.333333	213
0.666667	96
1.000000	654

**Table 8:** Distribution of dataset into training, validation, and testing sets.

Category	Intact	Defected
Training (70%)	725 (36.25%)	675 (33.75%)
Validation (20%)	207 (10.35%)	193 (9.65%)
Testing (10%)	103 (5.15%)	97 (4.85%)

**Table 9:** Summary of dataset collection, preprocessing, and annotation.

Aspect	Description
Data collection	2,000 EL images (monocrystalline & polycrystalline).
Labeling method	Images labeled into 0%, 33%, 67%, 100% defect probability.
Image preprocessing	Converted to 8-bit grayscale, and normalized for lighting.
Contrast adjustment	Histogram equalization applied to standardize brightness and visibility.
Data augmentation	Random flips, rotation, cropping, zoom, and Gaussian noise (via PyTorch).
Standardization	Pixel intensity scaled to [0, 1]; orientation and framing standardized.
Class imbalance	Balanced using oversampling and class-weighted “BCEWithLogitsLoss”.

### Calculation

**Fig. 14:**
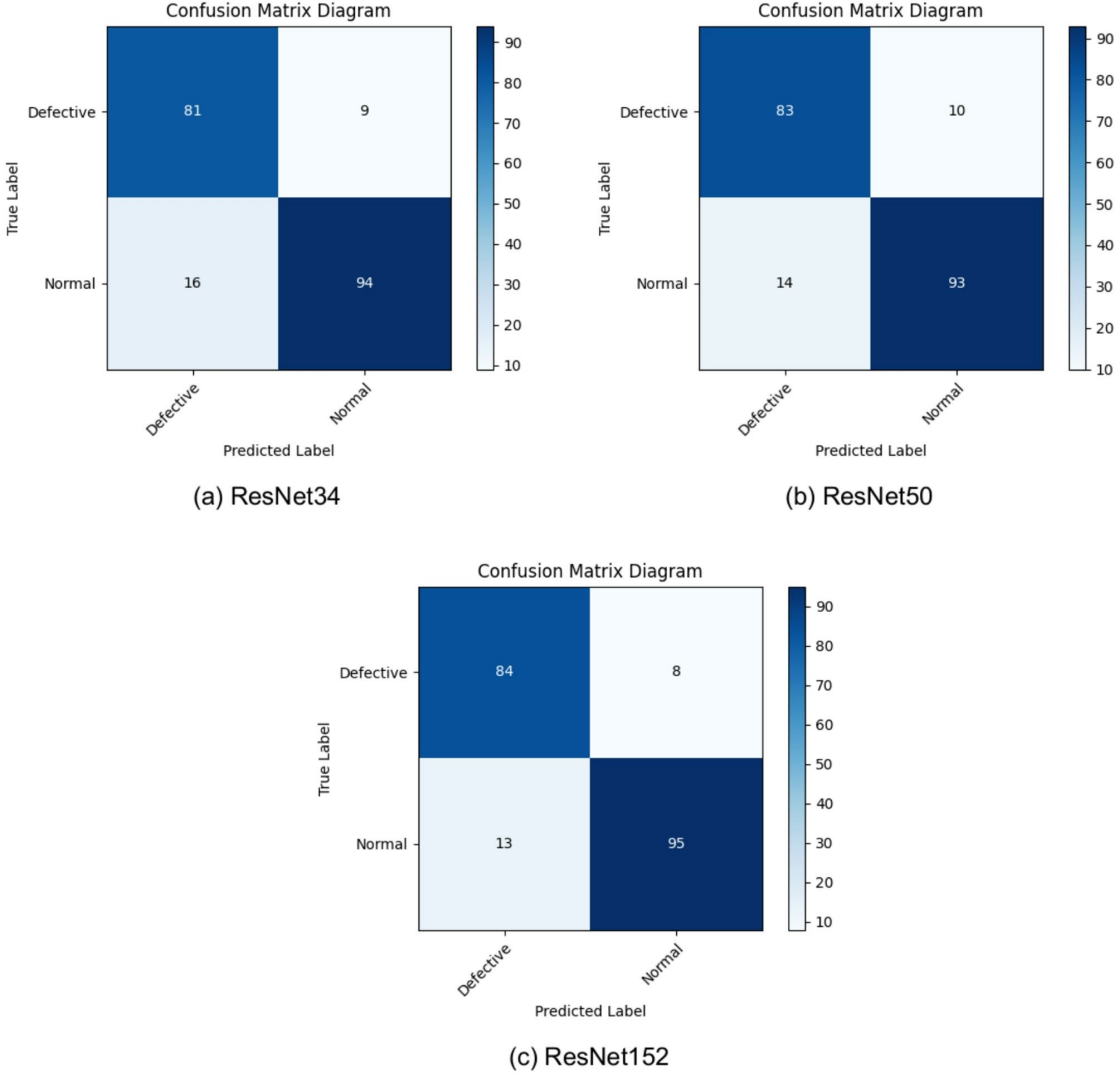
Confusion matrix diagrams for PV panel crack detection: (a) ResNet-34, (b) ResNet-50, (c) ResNet-152 architectures.

The research utilized a confusion matrix technique to assess the effectiveness of several ResNet models in identifying fractures in solar panels, as seen in Fig. 14. This method is crucial for comprehending the efficacy of the algorithms in categorizing faulty and standard panels. The performance indicators were calculated using precise formulas for precision, recall (sensitivity), and F1-score, offering a full evaluation of the correctness of the models, with these equations derived from the study^39^.

a) Precision:

This parameter (4) measures the accuracy of the model in correctly recognizing panels that are truly faulty. The calculation involves dividing the number of correctly predicted faulty instances (True Positives) by the total number of cases predicted as defective (the sum of True Positives and False Positives). Mathematically, precision is quantified or represented as follows:

4 $$\:Precision=\:\frac{\text{T}\text{P}}{\text{T}\text{P}+\text{F}\text{P}}$$

b) Recall:

Recall, sometimes referred to as sensitivity (5), quantifies the model’s capacity to accurately detect all true defective cases. The term ‘True Positive Rate’ refers to the proportion of correctly predicted faulty instances (True Positives) to the total number of defective cases, which is the sum of True Positives and False Negatives. The recall formula is:

5 $$\:Recall=\:\frac{\text{T}\text{P}}{\text{T}\text{P}+\text{F}\text{N}}$$

c) F-Score:

While accuracy and recall yield important information, their evaluation in isolation may not furnish a whole assessment of the model’s performance. The F-score, often known as the F1 score (6), resolves this issue by amalgamating precision and recall into a unified statistic. The harmonic mean of precision and recall is used to balance the two measures and provide a comprehensive evaluation. The F1 score is computed using the following equation:

6 $$\:{F}_{1}=\frac{2\times\:\text{R}\text{e}\text{c}\text{a}\text{l}\text{l}\times\:\text{P}\text{r}\text{e}\text{c}\text{i}\text{s}\text{i}\text{o}\text{n}}{\text{P}\text{r}\text{e}\text{c}\text{i}\text{s}\text{i}\text{o}\text{n}+\text{R}\text{e}\text{c}\text{a}\text{l}\text{l}}$$

### Code availability

The custom code for the ResNet-based crack detection framework is included as supplemental material to this research paper. This extensive codebase comprises modules for data preparation (histogram equalization, normalization), dataset partitioning (70%/20%/10% train/validation/test), and PyTorch implementations of customized ResNet34/50/152 architectures tailored for grayscale EL image processing. The training pipeline includes class-weighted loss functions, real-time augmentation (random flips, rotations, cropping), TensorBoard logging, and early termination based on F1-score optimization. The code facilitates deployment flexibility by enabling the export of trained models to ONNX format. All experiments detailed in Tables 5 and 10 may be replicated utilizing the setup settings integrated inside the script. The autonomous implementation guarantees complete repeatability of our methods without external dependencies.

## Results

This study primarily investigates two primary forms of defects in solar cells: (1) Microfractures and macrofractures (Cracks): These fractures can significantly differ in size, from minuscule, nearly undetectable microfractures to extensive macrofractures that encompass the whole cell. Fractures within a cell do not invariably affect its operation, as the electrical connection frequently persists even in regions exhibiting breaks. (2) Dormant areas: Usually resulting from cellular fractures are these ones. A part of the cell may become electrically separated due to fracture-induced disconnection, therefore reducing its total power output. This phenomenon directly affects the efficiency of cells. Although fractures can cause inactive areas sometimes, the method handles both defect types as separate entities. Regardless of its activity level, a cell is categorized as “cracked” just based on its outward display of fractures.

The findings of the investigation, presented in Table 10, indicate little difference among ResNet34, ResNet50, and ResNet152 in detecting fractures in solar panels. This assessment is performed by analyzing a dataset that comprises 10% of the total dataset. The collection comprises 103 complete images and 97 faulty images. The F1-Scores obtained were 86.63% for ResNet34, 87.37% for ResNet50, and 88.89% for ResNet152. The findings underscore that the incremental improvements in precision achieved with advanced ResNet models are not significantly justified by the additional computational resources and time required. Consequently, when evaluating the trade-off between computational efficiency and accuracy, the ResNet34 architecture stands out as the optimal choice. The confusion matrix diagrams are illustrated in Fig. 14. Exhibit the efficacy of ResNet-34, ResNet-50, and ResNet-152 in balancing processing speed, resource efficiency, and accuracy. This is especially apparent when evaluating the marginal accuracy improvements of higher-tier models relative to their augmented computing requirements. This comparative visualization reinforces the rationale for employing ResNet-34 in practical applications where efficiency and accuracy have equal significance.

**Table 10:** Performance comparison of ResNet architectures on PV panel crack detection.

ResNet Type	Recall	Precision	F1-Score
ResNet34	90.00%	83.51%	86.63%
ResNet50	89.25%	85.57%	87.37%
ResNet152	91.30%	86.60%	88.89%

ResNet34 offers a practical solution for real-world PV inspection due to its balance of accuracy and computational efficiency. Its moderate size enables deployment on edge devices like NVIDIA Jetson or Raspberry Pi with accelerators. The model delivers fast inference, supporting near real-time performance. When integrated with UAV platforms, it enables automated, large-scale PV monitoring, significantly reducing inspection time and cost. Overall, ResNet34’s efficiency makes it ideal for edge-based solar panel fault detection, Table 11 presents a general comparison between ResNet34, ResNet50, and ResNet152 models.

**Table 11:** General architectural comparison of ResNet34, ResNet50, and ResNet152 models.

Factor	ResNet34	ResNet50	ResNet152
Depth	34 layers	50 layers	152 layers
Architecture type	Standard residual blocks	Bottleneck residual blocks (1 × 1, 3 × 3, 1 × 1)	Bottleneck residual blocks
Parameters	~ 21.8 million	~ 25.6 million	~ 60.2 million
Speed vs. accuracy	Fastest; lower accuracy	Balanced speed and accuracy	Slowest; highest accuracy
Overfitting risk	Low; suitable for smaller datasets	Moderate; may require regularization	Higher; benefits from data augmentation
Hardware demands	Low; suitable for CPUs and low-end GPUs	Moderate; requires mid-range GPUs	High; needs high-end GPUs or TPUs

Although all three models examined—ResNet34, ResNet50, and ResNet152—obtained exceptional F1-scores, ResNet34 demonstrated the most advantageous equilibrium between computational efficiency and performance in the comparative assessment of ResNet architectures. ResNet34 was able to maintain a competitive F1-score of 86.63% and a high recall of 90% despite its comparatively simpler design. This ensured the efficient identification of defective panels and reduced the likelihood of unnoticed errors. This performance is attributed to its ability to effectively manage the precision-recall trade-off; it maintains sensitivity while delivering sufficient precision (83.51%). However, the computational burden of deeper models, such as ResNet50 and ResNet152, was significantly increased, rendering them unsuitable for real-time or edge deployment, because they need more time than ResNet34, despite the minor accuracy improvements they provided. Error analysis revealed that most misclassifications were related to minute microfractures and cell edge flaws, usually under low contrast or noisy settings.

Despite the high accuracy and computational efficiency of the proposed ResNet-based crack detection technique, some limitations still exist. Of course, the data dictionary is limited to the available images and may not cover all real world scenarios due to rare or complex defect patterns despite best attempts at augmentation and balancing. Finally, environmental and operational conditions like different illumination and noise within the EL images still influence the detecting accuracy. As such, overcoming these limitations is important with respect to the research targets, as it not only encourages progression towards next-generation datasets to make reliable approaches that will work in much broader conditions, a key aspect necessary for transferring the approach to an industrial framework and thus expects to see a trend of target predominance in future endeavors, focusing on diversity and robustness when working with multiple items.

Identifying defects in PV systems is essential for preserving their efficiency, guaranteeing stable energy output, and prolonging their operational longevity. Timely identification of faults mitigates energy losses, lowers maintenance expenses, and averts more damage to solar modules. PV systems are essential for harnessing clean, renewable solar energy, propelling worldwide initiatives to diminish emissions and attain a sustainable future^40–42^. Advancing research in PV technology will further augment its contribution to climate change mitigation and energy security enhancement.

## Conclusion

This study focuses on the domain of PV panel health monitoring, with a special emphasis on the identification of fractures through the utilization of modern image processing techniques. The study utilized an extensive dataset that included a wide variety of PV modules, consisting of a total of 44 modules. In both cases, the dataset was balanced between an 18-monocrystalline and a 26-polycrystalline module. This enables a comprehensive analysis of the classification capability on all kinds of solar panels with different types of cracks. A core part of the methodology is the use of ResNet architectures to detect cracks. The study, which looked at how well ResNet-34, ResNet-50, and ResNet-152 models worked, showed that as the models got more complicated, their accuracy kept going up. The F1 scores for ResNet-34, ResNet-50, and ResNet-152 were 86.63%, 87.37%, and 88.89%, respectively. Nevertheless, the enhancements in precision achieved through the use of more sophisticated ResNet models were not significant enough to warrant the allocation of extra computing resources and effort. Therefore, the ResNet34 architecture is considered the most preferable option because of its ability to achieve an ideal equilibrium between computing efficiency and detection accuracy. The data in the study technique was divided into three distinct categories: training (70%), validation (20%), and testing (10%). In the future, the investigations will focus on improving and optimizing the precision and effectiveness of PV panel fracture detection. The intention is to conduct experiments by training and testing on various network sizes in order to further decrease the occurrence of erroneous detections. In addition, the forthcoming research will entail a thorough investigation of several network topologies and configurations, such as ResNet, to evaluate their influence on detection accuracy and the utilization of computing resources. To summarize, this research establishes a solid basis for employing image processing techniques to identify fractures in PV panels. It offers vital insights for ensuring the long-term functionality and upkeep of solar PV systems. The proposed strategy would priorities the enhancement of these detection techniques to achieve higher levels of precision and effectiveness, therefore making a substantial contribution to the dependability and durability of solar energy infrastructure.

## Future work

PV is essential for enabling the capture of clean renewable energy from the sun to further decarbonize our society into a more sustainable future, and increasing the focus on scientific research in the field of solar energy will make this field an effective field in the field of renewable energy. The accuracy and efficiency of our ResNet-based image processing method for fracture identification on PV panel needs to be further improved in the future research from several aspects. One approach is to include more sophisticated DL models, such as transformers or hybrid architectures, which might enhance detection accuracy while optimizing computing efficiency. Furthermore, augmenting the dataset to incorporate a wider array of intricate fracture patterns and multiple PV technologies will enhance the model’s robustness under diverse settings. A further prospective study domain is the implementation of real-time crack detection algorithms capable of functioning effectively on edge devices. This may provide expedited on-site inspections, eliminating the necessity for centralized data processing, which is essential for extensive solar systems. Integrating these models with UAVs for automated inspection and monitoring might enhance the efficiency of PV panel maintenance. Furthermore, integrating environmental and operational data, like temperature, humidity, and irradiance levels, into the detection algorithms might yield a more thorough comprehension of crack advancement and its effect on panel performance. This would provide predictive maintenance, thereby decreasing the probability of expensive downtime. Future research must prioritize the optimization of models for lower-end hardware, facilitating the implementation of enhanced crack detection in areas or facilities with constrained computing capabilities, therefore advancing the worldwide initiative for accessible and sustainable solar energy solutions.

## Electronic supplementary material

Below is the link to the electronic supplementary material.

Supplementary Material 1

## Data Availability

All data generated or analysed during this study are included in this published paper. The custom code is provided in the supplementary materials. Additional data supporting the findings of this study are available from the corresponding author upon reasonable request.

## Abbreviations

BIPV: Building-Integrated Photovoltaic
BPNN: Back Propagation Neural Network
CNN: Convolutional Neural Network
CUDA: Compute Unified Device Architecture
CV: Computer Vision
DL: Deep Learning
EL: Electroluminescence
FLOPs: Floating Point Operations per Second
GANs: Generative Adversarial Networks
GPU: Graphics Processing Unit
IR: Infrared Radiation
ONNX: Open Neural Network Exchange
PID: Potential Induced Degradation
PL: Photoluminescence
PV: Photovoltaic
RF: Random Forest
ResNet: Residual Network
RGB: Red Green Blue
SEM: Scanning Electron Microscopy
SMOTE: Synthetic Minority Oversampling Technique
ST: Synchronized Thermography
SVM: Support Vector Machine
UAVs: Unmanned Aerial Vehicles
UV: Ultraviolet
